# Family predictors of physical activity change during the COVID-19 lockdown in preschool children in Germany

**DOI:** 10.1007/s10865-022-00382-7

**Published:** 2022-12-17

**Authors:** Franziska Beck, Steffen C. E. Schmidt, Alexander Woll, Anne K. Reimers

**Affiliations:** 1 Department of Sport Science and Sport, Friedrich-Alexander-Universität Erlangen-Nürnberg, Gebbertstraße 123B, 91052 Erlangen, Germany; 2 Institute of Sport Science & Sport, Karlsruher Institute of Technology, Engler-Bunte-Ring 15, 76131 Karlsruhe, Germany

**Keywords:** Preschool, COVID-19, Physical activity, Family

## Abstract

The COVID-19 pandemic is associated with crucial changes in children’s daily life including their physical activity (PA) and screen time (ST). Among preschool children, the family represents an important factor for sufficient PA levels by being the gatekeeper for PA. Thus, the aim of this study was to investigate the influence of the family environment, specifically SES, parental support, and having siblings on COVID-19-related changes of PA and ST behavior in 317 (170 boys, 147 girls) German preschool children using longitudinal data. Our results indicate a decline in total amount of sports-related PA, an increase in outdoor play, as well as an increase in leisure ST in preschool children. The changes in total amount of PA differed between children with different levels of parental support as well as in dependence on having siblings. Furthermore, levels of outdoor play and ST in preschool children were influenced by environmental factors like having access to their own garden. We conclude that the family environment (parental support as well as physical environment) is highly relevant for PA and ST levels in preschool children. To provide every child with PA opportunities during potential future lockdowns, restriction policies should be adapted and parents need sophisticated information about the importance of their support and thus the PA levels of their children.

## Introduction

In mid-December 2019, the first cases of the new Coronavirus (SARS-CoV-2) causing respiratory disease (COVID-19) in humans were reported from hospitals in Wuhan, China (Wu et al., 2020; Zhou et al., 2020). Due to the global spread of the virus in the subsequent weeks and months, the rising number of cases, and the high mortality, particularly among the elderly, the WHO declared COVID-19 as a global pandemic on March 11^th^, 2020 (World Health Organization, 2020) and measures to slow down the spread of the virus were implemented. The federal states of Germany closed preschools for children aged 3–5 years, schools, sports clubs, gyms, and other leisure institutions relevant to children’s and adolescents’ organized physical activity (PA) in March 2020. Furthermore, the government enforced physical distance measures and contact restrictions, allowing no more than two people from different households to meet in public space (Press and Information Office of the Federal Government, 2020a). Practicing organized sports was impeded due to the closing of public sports facilities and sports clubs. However, non-organized sports like going for a walk or playing outside remained allowed if done alone or with people from the same household (Press and Information Office of the Federal Government, 2020b). The lockdown was imposed until 3^rd^ May 2020 (Press and Information Office of the Federal Government, 2020c).

The COVID-19 outbreak and the following mitigation policies, especially the closure of preschools and home office for parents, resulted in crucial changes in children’s daily life and their movement behaviors (PA, sedentary behavior including screen time (ST)) (Bates et al., 2020; Chen et al., 2020; Guan et al., 2020; Hall et al., 2020). These policies foresee a revolution of one’s habits and lifestyle including the possibility to remain physically active during a forced isolation by adapting the movement and PA behavior (e.g., unstructured PA instead of organized sports opportunities, online PA offers for children) (Chirico et al., 2020). This is important as regular PA and low sedentary time are associated with numerous health benefits in childhood and adolescence (Poitras et al., 2016). In this context, we would like to differentiate PA and sedentary behavior as this explains why we assessed PA as well as ST. PA is defined as any bodily movement produced by skeletal muscles that requires energy expenditure. Thus, PA refers to all movement including during leisure time, for transport to get to and from places, or as part of a person’s work (Caspersen et al., 1985). On the other hand, sedentary behavior is different from being physically inactive, as it is possible to meet PA guidelines and still spend a large amount of time sitting. Thus, these two behaviors do not directly displace each other (Pearson et al., 2014) and are both included in the present study. In particular, as a result of regular PA as well as of reduced sedentary time, children improve their cardiovascular (Ekelund et al., 2012) and cardiometabolic (Ekelund et al., 2012; Poitras et al., 2016) health and reduce their risk of obesity (Committee, 2008; Hong et al., 2016; World Health Organization, 2009). Additionally, PA is associated with better mental health in children (Biddle et al., 2011) and adolescents (Rodriguez-Ayllon et al., 2019). Especially for preschoolers, commonly including children aged 2–5 years who are not old enough to go to a formal school, PA and bodily movement are also needed for healthy growth and cognitive, social, and emotional development of the child (Berk, 2013; Poitras et al., 2016). Furthermore, PA in early childhood PA is tracking through youth into adulthood and therefore contributes to an active lifestyle throughout one's lifespan (Telama et al., 2014).

Even if there is a lot of knowledge about the benefits of PA, research on PA and sedentary behavior during the COVID-19 pandemic indicated predominantly PA decreases (Aguilar-Farias et al., 2020; Carroll et al., 2020; Clarke et al., 2021; Guan et al., 2020; Jauregui et al., 2021; Kracht et al., 2021; Lopez-Bueno et al., 2020) whereas sedentary time including ST increases (Aguilar-Farias et al., 2020; Jauregui et al., 2021; Kracht et al., 2021; Lopez-Bueno et al., 2020) in preschool children compared to pre lockdown levels. Existing COVID-19 studies were cross-sectional studies using retrospective pre-COVID-19 data and considered only slightly differences in PA changes related to the social environment, even if PA levels differ between social variables as can be seen in pre-lockdown data among preschool children (Reimers et al., 2019). Data gathered before the pandemic showed that preschool children in Germany reduced their PA in unorganized activities from about 49 min per week (2003–2006) to 36 min per week (2009–2012) and 24 min per week (2014–2017) (Schmidt et al., 2020b; Schmidt et al. 2017) but slightly increased their organized sports activities. Thus, it would be quite interesting to see the changes of unorganized and organized PA as organized sports opportunities could not take place during the lockdown. Studies before the pandemic also indicated that the support of their family members seem to play an important role in promoting opportunities for PA among preschool children by being the gatekeeper for PA (Bingham et al., 2016; Gariguett et al., 2017; Hinkley et al., 2008; Loprinzi et al., 2010; Mitchell et al., 2012; Schmutz et al., 2017). Parental support contains different types, but there is no completely agreed-upon structure of the components in the literature (Rhodes et al., 2019). However, typically it includes instrumental or logistic support (e.g. transportation to activities), informational support (e.g. importance of PA within the family), emotional support (e.g. parental interest in child's PA), and companionship (e.g. parent and child doing PA together) (Pyper et al., 2016; Uchino, 2009). Due to the closure of preschools during the pandemic, preschool children require greater parental support which may mean managing and supervising children's activities and behaviors was more challenging for these families, particularly if parents were working (Prime et al., 2020). These circumstances are also reflected in PA levels in preschool children measured within a qualitative study: it was concluded that parental support for children's PA during the pandemic was easier when one parent was not working and therefore, the children of non-working parents accumulated higher PA levels than children whose parents both were working (Clarke et al., 2021). Furthermore pre-pandemic research suggested differences in children’s PA and ST levels related to their parents’ educational level and socioeconomic status (SES) with higher PA and lower ST in children from families with higher SES (Aguilar-Farias et al., 2020; Schmidt et al., 2021; Tandon, Zhou, Sallis, et al., 2012). Interestingly, during the pandemic, children whose parents are working from home and have a higher income or SES, tend to restrict their children's PA more, while at the same time may provide more opportunities to engage in ST (Aguilar-Farias et al., 2020; Clarke et al., 2021; Jauregui et al., 2021). A further social variable that was associated with higher PA levels before the pandemic is having someone to play with (Sigmund et al., 2021). In times of restrictions, siblings seem to increase the likelihood to be physically active. Thus, children having siblings were significantly more active during the lockdown than children without siblings (Aguilar-Farias et al., 2020; Clarke et al., 2021; Jauregui et al., 2021; Pombo et al., 2020). Lastly, outdoor opportunities and access to green spaces are associated with preschool children's PA levels (Benjamin-Neelon et al., 2019; Gray et al., 2015). However, with the closure of public green spaces, it was difficult for children to be physically active outdoors, especially if families haven’t their own garden. Data confirmed this relationship by indicating that children living in apartments without access to outdoor space revealed lower PA levels compared with children in houses or greater living areas (Aguilar-Farias et al., 2020; Clarke et al., 2021; Jauregui et al., 2021; Okely et al., 2021).

However, while research regarding changes in PA and ST levels in children and adolescents exists, only few studies investigated the influence of COVID-19 lockdown on preschool children. In particular, there is, until now, no study for Germany focusing exclusively on preschool children. Furthermore, only a few of the existing studies investigated changes from pre to during pandemic PA and ST levels by taking social variables like the family environment into account (Aguilar-Farias et al., 2020; Clarke et al., 2021; Jauregui et al., 2021). In addition, most of the previous COVID-19 studies are cross-sectional and used retrospective data for analysis of the changes.

Therefore, the aim of the present study is to investigate the influence of the family environment, specifically SES, parental support, garden ownership, and having siblings on COVID-19-related changes in PA and ST behavior in German preschool children aged 4–5 years using longitudinal data.

## Material and methods

The STROBE guidelines (Vandenbroucke et al., 2007) guided the writing of the current manuscript.

### Study design

The Motorik-Modul (MoMo) Study is a nationwide longitudinal study on PA, motor performance, and health in children and adolescents from Germany and has been started in 2003 (Wagner et al., 2014; Woll et al., 2021). Until now, besides the MoMo baseline study (2003–2006), three further measurement time points (waves) have been taken with Wave 3 starting in August 2018. This wave was planned to be finished in June 2020. Due to the COVID-19 situation and the related lockdown in Germany, the study had to be interrupted in March 2020. Alternatively, a MoMo COVID-19 lockdown study was conducted from 04/20/2020–04/30/2020. All participants of the regular MoMo wave 3 were asked to take part in this COVID-19 lockdown study and to answer an online questionnaire about their PA behavior during the lockdown. The individual time span between the two measurements of MoMo wave 3 and the MoMo COVID-19 lockdown study ranged from 1 to 27 months.

The study was conducted according to the Declaration of Helsinki. Ethics approval was obtained by Charité Universitätsmedizin Berlin ethics committee, by the University of Konstanz, and the ethics committee of the Karlsruhe Institute of Technology (KIT) (Wave 2 and 3, a positive ethics vote was given on 23 September 2014 by the ethics committee of the KIT). The Federal Commissioner for “data protection” and “freedom of information” and the Federal Office for the Protection of Data were informed about the study and approved it. Children and adolescents participated voluntarily and every participant received a gift worth 20€. They provided written informed assent and (parental) consent. This paper reports pre-COVID-19 data that was derived from the third follow-up of the MoMo Study (the interrupted wave 3) and data from the COVID-19 lockdown study (during-COVID).

### Participants

MoMo Wave 3 participants (pre-COVID-19) were selected based on a nationwide multistage sampling approach with two evaluation levels to ensure the best representativeness possible (Schmidt et al., 2020a). First, 167 sampling units were systematically selected from an inventory of German communities which assesses the level of urbanization and geographical distribution (Lampert et al., 2014). Second, an age-stratified sample based on the official population registers was drawn and invited to the study.

Overall, 2843 participants of MoMo Wave 3 (preliminary response 25.2%) were contacted for the within-COVID-19 assessment, and data from 1711 participants (4–17 years old) were gathered. A total longitudinal response of 63.6% was achieved. The reasons for non-participation are unknown due to data protection regulations. In this paper, we analyzed the data of 317 preschool children (mean age: 5.04 ± 0.55) (see Table 1).

**Table 1:** Sample characteristics

		4-year-olds	5-year-olds	Total
Total [n]		150	167	317
Boys [n (%)]		79 (52.7%)	91 (54.5%)	170 (53.6%)
Girls [n (%)]		71 (47.3%)	76 (45.5%)	147 (46.4%)
Age pre COVID (years; M ± SD)		4.54 ± 0.28	5.50 ± 0.28	5.05 ± 0.55
SES [n (%)]	Low	16 (11.0%)	20 (12.3%)	36 (11.7%)
	Mid	108 (74.0%)	118 (72.8%)	226 (73.4%)
	High	22 (15.1%)	24 (14.8%)	46 (14.9%)
Garden ownership (yes) [n (%)]		121 (80.7%)	149 (89.2%)	270 (85.2%)
Siblings (yes) [n (%)]	Active	81 (55.1%)	91 (54.5%)	172 (54.8%)
	In active	43 (29.3%)	43 (25.7%)	86 (27.4%)
	No siblings	23 (15.6%)	33 (19.8%)	56 (17.8%)

*M* mean;* SD* standard deviation

### Data collection

Within the MoMo wave 3 data collection participants were invited to examination rooms within a distance of 15 km from their homes and answered the MoMo PA questionnaire (MoMo-PAQ) (Schmidt et al., 2016) on laptops. For preschool children, parents filled in the questionnaire (proxy-report). For the Follow-up survey during the lockdown (during COVID) parents answered the questionnaire through a link sent to them via e-mail. Further information about data collection has been published elsewhere (Schmidt et al., 2020b).

### Measures

#### Demographics

Demographic data were based on information from MoMo Wave 3 data including age, sex, and a multitude of socioenvironmental and socioeconomic variables. For the present study, we included the SES (Lampert et al., 2014), as well as garden ownership. Individual SES was defined by the total household income per household member as well as the educational and professional status of the parents. Both parents were separately asked about their educational and professional status (Lampert et al., 2014). Preschool children with separated parents were assigned to the SES of the parent they lived with. The sum score was then categorized by quintiles of the whole MoMo Wave 3 sample into low (first quintile: < 9), medium (9–17), and high (fifth quintile: > 17) SES. Detailed information about this approach can be found elsewhere (Lampert et al., 2014).

#### Physical activity

To assess PA we used the MoMo-PAQ with sufficient reliability and validity (test–retest reliability: ICC ≤ 0.68) (Jekauc et al., 2013). Participants were asked about their sports activity in organized (sports club, school) and nonorganized leisure sports activities. Parents could then answer questions about frequency, duration, type, and intensity of their children engaging in each setting by proxy-report. Items were then combined in one index (= total amount of sports-related PA) that reflects the daily minutes with organized (school and sports clubs) and non-organized PA. Detailed information about the PA measure has been published elsewhere (Schmidt et al., 2020b).

Furthermore, participants were separately asked about minutes per day of non-organized playing outside (Schmidt et al., 2016).

#### Recreational screen time

ST of the preschool children was measured via proxy-reported ST behaviors by the parents. Parents were asked to report their preschoolers’ time spent watching TV, playing games on any device, and using the internet for recreational use separately on weekdays and weekends using a 7-point scale including (almost) never, 15 min per day, 30 min per day, 1 h per day, 2 h per day, 3 h per day, and 4 h per day (Mathers et al., 2009). ST was summed up to gain a total amount of recreational ST in minutes per day.

#### Social support

Social support was assessed by parental support as well as having an (active) sibling. The parental support scale includes eight items and could be answered on a four-point rating scale (1—“not at all” to 4—“very important”) (Jekauc et al., 2013). Higher scores indicated higher perceptions of social support. The scale was divided into four subscales with two items each. Parents then reported (proxy) following questions for their preschool child: emotional support (‘How important is sport in your family?’ and ‘How important is it to your parents that you do sports?’), instrumental support (‘How often are you driven to sports venues by your parents?’ and ‘Do your parents support you in your sports activity?’), informational support (‘How interested are your parents in your sport?’ and ‘How often is your sport a topic of conversation in your family?’), and parental companionship (‘Do your parents engage in sports with you?’ and ‘How often do your parents watch you doing sports?’) (Reimers et al., 2012). Furthermore, participants were asked “Is at least one of your siblings physically active on a regular basis?” and this could be answered by “yes”, “no” or “I don’t have siblings”.

### Data analysis

All statistical tests were conducted using IBM SPSS 27 (IBM Corporation, Armonk, NY). Descriptive statistics for age, gender, SES, as well as garden ownership, and sibling were calculated and separated for 4- and 5-year-olds.

Separate single factor repeated measurement ANOVAs were used to analyze the influence of gender, social support through the family, SES, and the fact whether the child has access to an own garden on the change in PA and ST to prevent overparameterization. Statistical significance was set to p < 0.05.

## Results

Overall, 170 boys (53.6%) and 147 girls (46.4%) were included in the present study (Table 1). Overall, the mean age pre COVID-19 was 5.05 ± 0.55 years. Most of the preschool children had a mid SES (73.4%) and access to their own garden (85.2%). Regarding siblings, only 17.8% had no siblings whereas 54.8% had an active sibling.

Table 2 shows influencing factors on the change in the total amount of sports-related PA from before to during the COVID-19 lockdown. First of all, the total amount of sports-related PA did not significantly decrease from pre to peri lockdown in boys and girls (F_1,305_ = 1.59; p = 0.21; p.Eta^2^ = 0.005) with no significant differences between boys and girls. Parental support (emotional, informational instrumental, and companion), as well as siblings, influenced the total amount of sports-related PA in preschool children. These main effects revealed significance with medium effect sizes. Related to an interaction, descriptive data indicated a greater reduction of sports in preschool children with lower parental support and having an inactive sibling. However, rmANOVAs did not reveal significance for the time*factor interaction. SES as well as having an own garden did not influence the total amount of sports-related PA in preschool children.

**Table 2:** Factors influencing the total amount of sports-related PA in 4–5 old children before and during the COVID-19 lockdown in Germany (MoMo Study)

Total amount of sports-related PA [minutes per week]							
Age 4–5 years	Descriptives				rmANOVA		
Factor	Value	n	Pre (M ± SD)	Peri (M ± SD)	Time	Factor	Time*factor
Gender	Boys	170	117.4 ± 79.4	98.8 ± 204.4	F_1,305_ = 1.59 p = 0.21 p.Eta^2^ = 0.005	F_1,305_ = 0.40 p = 0.53 p.Eta^2^ = 0.001	F_1,305_ = 0.06 p = 0.80 p.Eta^2^ < 0.001
	Girls	147	122.6 ± 98.0	110.1 ± 201.3			
Parental support: emotional	Low (< 2.5)	35	93.8 ± 91.1	26.7 ± 91.6	F_1,302_ = 3.46 p = 0.06 p.Eta^2^ = 0.011	F_2,302_ = 7.69 p < 0.01 p.Eta^2^ = 0.048	F_2,302_ = 1.28 p = 0.28 p.Eta^2^ = 0.008
	Med (2.5–3)	157	111.8 ± 80.8	95.9 ± 183.6			
	High (> 3)	113	140.2 ± 93.8	139.5 ± 243.8			
Parental support: informational	Low (< 2.5)	34	81.1 ± 77.8	30.9 ± 77.3	F_1,300_ = 2.73 p = .10 p.Eta^2^ = .009	F_2,300_ = 9.14 p < 0.01 p.Eta^2^ = 0.057	F_2,300_ = 0.48 p = 0.62 p.Eta^2^ = 0.003
	Med (2.5–3)	148	108.9 ± 82.7	94.0 ± 202.3			
	High (> 3)	121	146.2 ± 91.3	136.3 ± 223.2			
Parental support: instrumental	Low (< 2.5)	62	67.4 ± 61.6	45.6 ± 156.1	F_1,298_ = 2.27 p = 0.13 p.Eta^2^ = 0.010	F_2,298_ = 14.5 p < 0.01 p.Eta^2^ = 0.089	F_2,298_ = 1.55 p = 0.21 p.Eta^2^ = 0.010
	Med (2.5–3)	112	125.3 ± 94.2	82.5 ± 179.1			
	High (> 3)	127	142.8 ± 83.7	148.8 ± 232.0			
Parental support: companion	Low (< 2.5)	52	99.1 ± 79.0	67.3 ± 178.2	F_1,300_ = 2.78 p = 0.10 p.Eta^2^ = 0.009	F_2,302_ = 4.06 p = 0.02 p.Eta^2^ = 0.026	F_2,300_ = 0.50 p = 0.61 p.Eta^2^ = 0.003
	Med (2.5–3)	182	113.1 ± 75.3	106.2 ± 204.9			
	High (> 3)	69	157.6 ± 113.8	125.2 ± 216.7			
Siblings	Active	168	126.7 ± 91.9	117.2 ± 217.9	F_1,301_ = 0.85 p = 0.36 p.Eta^2^ = 0.003	F_2,301_ = 3.64 p = 0.03 p.Eta^2^ = 0.024	F_2,301_ = 1.99 p = 0.14 p.Eta^2^ = 0.013
	Not active	83	109.3 ± 89.6	58.7 ± 129.5			
	No siblings	53	115.8 ± 73.5	137.9 ± 239.7			
Family SES	Low (< 9)	35	119.9 ± 120.8	82.9 ± 168.0	F_1,295_ = 1.62 p = 0.20 p.Eta^2^ = 0.005	F_2,295_ = 0.56 p = 0.57 p.Eta^2^ = 0.004	F_2,295_ = 0,21 p = 0.81 p.Eta^2^ = 0.001
	Med (9–17)	219	116.5 ± 81.9	104.9 ± 203.5			
	High (> 17)	44	135.7 ± 96.9	119.2 ± 234.0			
Garden ownership	No	45	124.5 ± 87.9	85.2 ± 145.5	F_1,305_ = 2.18 p = 0.14 p.Eta^2^ = 0.007	F_1,305_ = 0.20 p = 0.66 p.Eta^2^ = 0.001	F_1,305_ = 0.64 p = 0.43 p.Eta^2^ = 0.002
	Yes	262	118.9 ± 88.4	107.2 ± 211.1			

*M* mean;* SD* standard deviation

Table 3 shows the factors influencing playing outside. There was a significant main effect for time indicating a significant increase of minutes per day playing outside (F_1,313_ = 49.7; p < 0.01; p.Eta^2^ = 0.137). This did not differ between boys and girls. Siblings did not influence the minutes per day playing outside in preschool children. In contrast to emotional, informational, and instrumental support, parental companionship showed a time-stable positive effect on playing outside (F_2,309_ = 3.15, p = 0.04, p.Eta^2^ = 0.020). Furthermore, having an own garden indicated a significant interaction effect with time and playing outside. Descriptive statistics revealed a larger increase in playing outside during the lockdown among preschool children who live in a house or apartment with a garden (F_1,313_ = 5.51, p = 0.02, p.Eta^2^ = 0.017).

**Table 3:** Factors influencing playing outside in 4–5 old children before and during the COVID-19 lockdown in Germany (MoMo Study)

Playing outside [minutes per day]							
Age 4–5 years	Descriptives				rmANOVA		
Factor	Value	n	Pre (M ± SD)	Peri (M ± SD)	Time	Factor	Time*factor
Gender	Boys	169	92.4 ± 57.3	131.8 ± 104.9	F_1,313_ = 49.7 p < 0.01 p.Eta^2^ = 0.137	F_1,313_ = 0.65 p = 0.42 p.Eta^2^ = 0.002	F_1,313_ = 0.31 p = 0.58 p.Eta^2^ = 0.001
	Girls	146	83.3 ± 58.1	129.5 ± 96.6			
Parental support: emotional	Low (< 2.5)	36	97.4 ± 60.1	142.8 ± 116.3	F_1,311_ = 35.7 p < 0.01 p.Eta^2^ = 0.103	F_2,311_ = 1.28 p = 0.28 p.Eta^2^ = 0.008	F_2,311_ = 0.39 p = 0.68 p.Eta^2^ = 0.002
	Med (2.5–3)	159	85.5 ± 60.4	123.4 ± 92.7			
	High (> 3)	119	88.8 ± 53.7	138.0 ± 106.2			
Parental support: informational	Low (< 2.5)	35	83.8 ± 56.9	127.0 ± 101.5	F_1,309_ = 33.6 p < 0.01 p.Eta^2^ = 0.098	F_2,309_ = 0.07 p = 0.93 p.Eta^2^ = 0.000	F_2,309_ = 0.12 p = 0.89 p.Eta^2^ = 0.001
	Med (2.5–3)	152	86.3 ± 60.5	131.8 ± 97.6			
	High (> 3)	125	90.2 ± 54.4	129.6 ± 100.7			
Parental support: instrumental	Low (< 2.5)	62	92.2 ± 73.3	131.2 ± 96.2	F_1,307_ = 41.6 p < 0.01 p.Eta^2^ = 0.119	F_2,307_ = 0.41 p = 0.67 p.Eta^2^ = 0.003	F_2,307_ = 0.05 p = 0.96 p.Eta^2^ = 0.000
	Med (2.5–3)	114	84.3 ± 51.6	125.5 ± 104.9			
	High (> 3)	134	89.4 ± 55.3	133.1 ± 95.5			
Parental support: companion	Low (< 2.5)	53	70.9 ± 54.0	116.6 ± 90.5	F_1,309_ = 41.6 p < 0.01 p.Eta^2^ = 0.119	F_2,309_ = 3.15 p = 0.04 p.Eta^2^ = 0.020	F_2,309_ = 0.34 p = 0.71 p.Eta^2^ = 0.002
	Med (2.5–3)	186	89.5 ± 58.0	128.0 ± 94.6			
	High (> 3)	73	96.1 ± 58.1	146.2 ± 114.4			
Siblings	Active	172	87.0 ± 55.5	134.7 ± 101.1	F_1,309_ = 37.1 p < 0.01 p.Eta^2^ = 0.107	F_2,309_ = 0.17 p = 0.84 p.Eta^2^ = 0.001	F_2,309_ = 0.72 p = 0.49 p.Eta^2^ = 0.005
	Not active	85	93.3 ± 68.3	124.2 ± 97.6			
	No siblings	55	82.6 ± 47.6	127.9 ± 107.3			
Family SES	Low (< 9)	36	80.1 ± 54.8	144.1 ± 95.2	F_1,303_ = 28.7 p < 0.01 p.Eta^2^ = 0.086	F_2,303_ = 0.12 p = 0.88 p.Eta^2^ = 0.001	F_2,303_ = 1.05 p = 0.35 p.Eta^2^ = 0.007
	Med (9–17)	225	87.8 ± 57.4	130.0 ± 101.7			
	High (> 17)	45	99.0 ± 63.5	131.4 ± 102.0			
Garden ownership	No	47	90.1 ± 62.4	99.0 ± 105.2	F_1,313_ = 11.6 p < 0.01 p.Eta^2^ = 0.036	F_1,313_ = 3.20 p = 0.07 p.Eta^2^ = 0.010	F_2,313_ = 5.51 p = 0.02 p.Eta^2^ = 0.017
	Yes	268	87.8 ± 57.0	136.3 ± 99.3			

*M* mean;* SD* standard deviation

Table 4 shows that leisure ST increased from pre to peri the lockdown in preschool children (F_1,314 = _204.5; p < 0.01; p.Eta^2^ = 0.394) with no main effect for gender. Furthermore, it can be seen that socioeconomic status (F_2,304_ = 3.20, p = 0.04, p.Eta^2^ = 0.021) as well as having an own garden (F_1,314_ = 3.88, p = 0.05, p.Eta^2^ = 0.012) had an influence on leisure ST in preschool children. Descriptive data indicated higher amounts of screen time with lower SES and not having an own garden. A small interaction effect can be seen for parental companionship (F_2,309_ = 3.11, p = 0.04, p.Eta^2^ = 0.020).

**Table 4:** Factors influencing leisure screen-time in 4–5 old children before and during the COVID-19 lockdown in Germany (MoMo Study)

Leisure screen-time [minutes per day]							
Age 4–5 years	Descriptives				rmANOVA		
factor	value	*n*	Pre (M ± SD)	Peri (M ± SD)	Time	Factor	Time*factor
Gender	Boys	170	52.7 ± 47.8	97.4 ± 64.8	F_1,314=_204.5 p < 0.01 p.Eta^2^ = 0.394	F_1,314_ = 0.65 p = 0.80 p.Eta^2^ = 0.003	F_1,314_ = 1.79 p = 0.18 p.Eta^2^ = 0.006
	Girls	146	51.4 ± 47.7	88.4 ± 64.2			
Parental support: emotional	Low (< 2.5)	36	63.1 ± 73.8	99.3 ± 63.5	F_1,311_ = 131 p < 0.01 p.Eta^2^ = 0.296	F_2,311_ = 0.81 p = 00.44 p.Eta^2^ = 0.002	F_2,311_ = 0.35 p = 0.71 p.Eta^2^ = 0.002
	Med (2.5–3)	159	53.5 ± 44.2	93.6 ± 63.1			
	High (> 3)	119	47.3 ± 42.0	91.0 ± 67.6			
Parental support: informational	Low (< 2.5)	35	58.6 ± 40.6	97.7 ± 51.3	F_1,309_ = 136 p < 0.01 p.Eta^2^ = 0.305	F_2,309_ = 0.26 p = 0.77 p.Eta^2^ = 0.002	F_2,309_ = 1.08 p = 0.34 p.Eta^2^ = 0.007
	Med (2.5–3)	152	52.6 ± 54.5	90.1 ± 66.5			
	High (> 3)	125	50.3 ± 40.7	96.6 ± 66.1			
Parental support: instrumental	Low (< 2.5)	63	55.9 ± 53.3	87.5 ± 65.3	F_1,307_ = 170 p < 0.01 p.Eta^2^ = 0.357	F_2,307_ = 1.95 p = 0.16 p.Eta^2^ = 0.012	F_2,307_ = 1.68 p = 0.19 p.Eta^2^ = 0.011
	Med (2.5–3)	114	57.0 ± 54.1	103.1 ± 69.0			
	High (> 3)	133	46.8 ± 38.6	88.4 ± 60.6			
Parental support: companion	Low (< 2.5)	53	63.8 ± 66.4	91.1 ± 64.0	F_1,309_ = 128 p < 0.01 p.Eta^2^ = 0.293	F_2,309_ = 1.70 p = 0.19 p.Eta^2^ = 0.011	F_2,309_ = 3.11 p = 0.05 p.Eta^2^ = 0.020
	Med (2.5–3)	186	52.3 ± 45.3	98.6 ± 68.5			
	High (> 3)	73	44.5 ± 35.8	82.4 ± 54.3			
Siblings	Active	172	47.9 ± 44.3	89.9 ± 59.5	F_1,310_ = 171 p < 0.01 p.Eta^2^ = 0.355	F_310_ = 1.08 p = 0.34 p.Eta^2^ = 0.007	F_2,310_ = 0.28 p = 0.76 p.Eta^2^ = 0.002
	Not active	85	57.8 ± 52.5	95.6 ± 65.1			
	No siblings	56	56.5 ± 50.0	100.1 ± 77.5			
Family SES	Low (< 9)	36	73.1 ± 63.8	110.9 ± 70.1	F_1,304_ = 115 p < 0.01 p.Eta^2^ = 0.274	F_2,304_ = 3.20 p = 0.04 p.Eta^2^ = 0.021	F_2,304_ = 0.60 p = 0.55 p.Eta^2^ = 0.004
	Med (9–17)	225	49.6 ± 45.8	88.7 ± 61.8			
	High (> 17)	46	48.1 ± 40.3	96.8 ± 68.5			
Garden ownership	No	47	64.6 ± 78.6	107.6 ± 75.9	F_1,314_ = 109 p < 0.01 p.Eta^2^ = 0.258	F_314_ = 3.98 p = 0.05 p.Eta^2^ = 0.012	F_1,314_ = 0.08 p = 0.78 p.Eta^2^ = 0.000
	Yes	269	49.9 ± 39.7	90.7 ± 62.2			

*M* mean;* SD* standard deviation

## Discussion

The aim of the present study was to investigate the influence of the family environment, specifically SES, parental support, garden ownership, and having siblings on COVID-19-related changes in PA and ST behavior in German preschool children using longitudinal data.

Our results show a non-significant decline in the total amount of sports-related PA in preschool children from pre to during the first lockdown of the COVID-19 pandemic. The non-significance could be explained by the great variance in preschool children with some children having 0 min of the total amount of sports-related PA per week while other preschool children reached 1000 min per week and more. Overall, during the first lockdown, restrictions like the closure of sports clubs, preschools as well as contact restrictions had a large impact on everybody's daily life and one’s opportunities for being active. Other studies investigating PA levels in preschool children also indicated a decrease in the total amount of sports-related PA (Aguilar-Farias et al., 2020; Carroll et al., 2020; Clarke et al., 2021; Guan et al., 2020; Jauregui et al., 2021; Kracht et al., 2021) and thus, confirmed our descriptive findings of decreasing total amounts of sports-related PA, even if data did not reach statistical significance. However, a study from Sweden found increases in PA levels in preschool children (Delisle Nystrom et al., 2020). Contrary to the other countries, Sweden did not close preschools, parks, or playgrounds, and organized sports continued. These findings from different countries highlighted the importance of preschools, playgrounds, and sports clubs for the accumulation of PA levels throughout the day.

We extended our study by focusing on different social influencing factors on the change of the total amount of sports-related PA in preschool children as especially for younger children families and their support seem to be associated with PA levels in preschool children as parents are the gatekeeper for PA (Bingham et al., 2016; Gariguett et al., 2017; Hinkley et al., 2008; Loprinzi et al., 2010; Mitchell et al., 2012; Schmutz et al., 2017). In this context, our data indicated an influence of parental support on preschool children's total amount of sport-related PA. Our descriptive data, that did not reach statistical significance, indicated that more parental support is associated with higher amounts of total sports activity pre-lockdown, and preschool children's sports levels during the lockdown did not strongly change when they received more parental support. Due to the closure of preschools during the pandemic, preschool children require larger parental support which may mean managing and supervising children's activities and behaviors was more challenging for these families, particularly if parents were working (Prime et al., 2020). In this context, an existing qualitative study concluded that parental support for children's PA during the pandemic was easier when one parent was not working and therefore, these children accumulated higher PA levels (Clarke et al., 2021; Pombo et al., 2020). Another explanation that should be taken into account refers to the function of modeling. PA levels in children can depend on the activity level of their parents as they play an important role as health behavior models (Sigmund et al., 2008).

Furthermore, the amount of sports-related PA in preschool children differed depending on having a(n) (active) sibling. Our data described statistically significant differences with the highest levels of sports in preschool children having an active sibling while having an inactive sibling is associated with the lowest levels of sports activity. Regarding the descriptive, not reaching statistical significance data, it can be seen that before lockdown, active siblings play fewer minutes outside with their siblings compared to non-active siblings (87.0 ± 55.5 vs. 93.3 ± 68.3). This could be explained by active siblings engaging more time in organized sports compared to playing with their siblings. During the lockdown no organized PA was allowed and active siblings could then be more engage in playing outside with their siblings (see Table 3). Existing COVID-19 literature showed that having someone to play with in times of restrictions, especially siblings, increases the likelihood to be physically active and thus, these children were significantly more active during the lockdown than children without siblings (Aguilar-Farias et al., 2020; Clarke et al., 2021; Jauregui et al., 2021; Pombo et al., 2020). Nevertheless, even if our data did not reach statistical significance, it seems important that the sibling is active too. Otherwise, PA levels decreased. Furthermore, having no sibling seems to be associated with a higher total amount of sports activity during the lockdown. This is slightly different from other studies indicating that children with siblings were more active during the lockdown than children without siblings (Aguilar-Farias et al., 2020; Clarke et al., 2021; Jauregui et al., 2021; Pombo et al., 2020). Nevertheless, these differences should be interpreted with caution because existing studies did not differentiate between active or inactive siblings.

Furthermore, the MoMo-PA questionnaire differentiates between different types of PA and therefore, we were able to analyze outdoor play separately. Our data indicated a significant increase in outdoor play across 4–5-year-olds in Germany with no differences between boys and girls. Non-essential institutions and sports clubs were closed (Press and Information Office of the Federal Government, 2020b), so children had more recreational time to engage in outdoor play during the lockdown. Data from the US highlighted the relevance of outdoor play during COVID-19 lockdown to stay physically active (Dunton et al., 2020). Studies from other countries revealed decreases in PA that can be explained by different policy restrictions. During the lockdown in China, for example, outdoor activities were not allowed (Chen et al., 2020). Similar results were seen in Spain where leaving the house was only allowed for work, grocery shopping, or doctor’s visit (Guan et al., 2020). In Sweden, the preschools remained open, and outdoor activities were allowed, so children showed an increase in time spent outdoors on weekdays as well as weekend days. Additionally, Swedish preschools changed their routines to have their children outside as much as possible (Delisle Nystrom et al., 2020). These examples support the assumption of a relationship between policy restrictions and outdoor play levels during pandemic (Beck et al., 2021).

Our data indicate significant differences in the levels of outdoor play between children receiving different levels of companionship from their parents. The increase in levels of outdoor play was greater in children whose parents often accompany their preschool children in their activities than in preschool children with less companionship support, as could be seen in the descriptive data. These not reaching statistical significance findings could be explained by the need for parental supervision and companionship for outdoor play in preschool children (Tandon et al., 2012a, 2012b). Furthermore, an interaction effect between an own garden and the lockdown related to outdoor play was significant. The results indicate a larger increase in preschool children who have access to their own garden. Due to the closure of recreational playgrounds, access to an own garden provides the opportunity for playing outside and this represents one of the strongest predictors for PA during the lockdown (Aguilar-Farias et al., 2020; Schmidt et al., 2021). In England, a qualitative study supported the importance of outdoor space for preschool children’s PA levels. Parents with no outdoor space described this as quite challenging: *‘We couldn't leave and there was nothing really you could do. And living in a block of flats, you can't be too noisy because you've got neighbours everywhere.’* (Clarke et al., 2021). These data demonstrated that having access to outdoor space is an important predictor for outdoor play and thus, PA levels in preschool children.

Complementary to a decrease in the total amount of sports-related PA, our data revealed a significant increase in leisure ST in preschool boys and girls from pre to during the lockdown in Germany. This finding is confirmed by further studies across various countries (Aguilar-Farias et al., 2020; Jauregui et al., 2021; Kracht et al., 2021; Lopez-Bueno et al., 2020). In particular, Lopez-Bueno et al. (2020) reported an increase of 132 min per day of screen exposure in Spanish 3–5-year-old children, and, preschool children in Chile doubled their ST during the lockdown (Aguilar-Farias et al., 2020). With higher time spent at home due to the restrictions, it was expected that screen exposure could reach higher levels than before the COVID-19 confinement. In this context, the COVID-19 pandemic has imposed digital platforms as this was the only mean to stay in contact with other people (Pandya & Lodha, 2021). Furthermore, the WHO stated to stay active by engaging in online sports offers or active video games (World Health Organization, 2020). This needs to be considered when interpreting the increasing ST in preschool children. Nevertheless, the SES as well as having an own garden seems to be associated with the change in ST. Our descriptive data revealed that the great differences in ST between low, medium, and high SES before the lockdown were getting smaller during the lockdown, even if the interaction effect doesn’t reach statistical significance. This is in line with another study indicating that especially preschool children from higher educated parents spent much time engaging in screen time during the lockdown because these parents had to work from home more frequently (Aguilar-Farias et al., 2020). This is interesting as, before the pandemic, children with more educated caregivers tended to engage with less time with screen-based devices. This may be partially explained as more educated caregivers may have to work from home, and this, in turn, may require the caregiver to use screens to entertain their child while working from home (Aguilar-Farias et al., 2020). Furthermore, higher income and thus higher SES is affected with garden ownership (Al-Dala'een, 2017). Our descriptive, not statistical significant results showed that preschool children living in an apartment or house with an own garden had lower levels of ST pre and during the lockdown. These children had more opportunities to spend recreational time outside and thus, less need to engage in ST (Clarke et al., 2021).

Undoubtedly, parental support as well as having active siblings play a key role in facilitating movement behaviors of preschoolers as we can see in our data. Nevertheless, some political actions during lockdowns may boost the process. Our findings reinforce the need for ensuring an activity-friendly environment for children at home and in surrounding areas to play. This is likely to promote these behaviors not only during the pandemic but also in the return to a “new normal” throughout all socioeconomic classes (Aguilar-Farias et al., 2020; Schmidt et al., 2021). Thus, decision-makers should seek options to facilitate outdoor recreational activities while preserving safety and physical distancing instructions. Furthermore, specific content for promoting PA through social media and national TV as a response to the pandemic could be installed as it was done in particular in Chile (Elige Vivir Sano Ministerio de Desarrollo Social y Familia Gobierno de Chile 2022).

### Limitations

Despite the comprehensive and longitudinal approach to investigating PA in preschool children during the COVID-19 lockdown in Germany, there are some limitations to our study. First, the representativeness of our longitudinal sample is limited because of the unforeseen lockdown during the collection of the pre-study sample which resulted in an interruption of the field research. Furthermore, the preliminary response rate of 25% also affected the representativeness of the present study. Second, the results of PA in Germany within the MoMo-Study are based on self-reports, respectively for preschool children, parents fulfilled the questionnaire (proxy-report). This could limit the validity of the answers as parents act as mediators and especially related to the social support scales answers could be influenced by social expectations. Furthermore, non-literary parents had problems to help their children at all. Nevertheless, the used questionnaire assessed different settings and types of PA and featured a higher focus on recall bias compared to unspecific questionnaires used in other studies (Delisle Nystrom et al., 2020; Lopez-Bueno et al., 2020). Third, as the present study is a natural experiment there is no control group and we can only assume that the changes in PA were caused by the lockdown. Due to the fact that we analyze longitudinal data from 4 to 5-year-olds before the lockdown and a full representative capture of Germany takes two years in our design, the mean age for the second measurement point during the lockdown exceeded the mean age pre-lockdown by almost exactly one year. Lastly, an increase in outdoor play can be explained by the weather during the COVID-19 lockdown and the questionnaire in April 2020 in Germany. It was untypically warm with a mean temperature of 10.4 °C (9.6 °C in 2019) and on average 292.4 sunshine hours (227.9 in 2019) (German Weather Service, 2019, 2020) which could have influenced the outdoor activities of children and their families.

## Conclusion

The results of the current study revealed that during the COVID-19 lockdown, the total amount of sports activity decreased while outdoor play, as well as recreational ST, increased in preschool children in Germany. Furthermore, our data indicated that especially for doing sports parental support and siblings seem to be important predictors for preschool children. In addition to this, the physical environment like having an own garden can positively influence health-related behaviors, especially outdoor play and ST in preschool children. To provide every child with PA opportunities during potential future lockdowns, restriction policies should be adapted and parents need sophisticated information about the importance of their support and thus the PA levels of their children.

## Data Availability

Data cannot be shared publicly because of strict ethical conditions with which study investigators are obliged to comply: The Charité/Universitätsmedizin Berlin ethics committee and the Federal Office for the Protection of Data explicitly forbid making the data publicly available because informed consent from study participants did not cover public deposition of data. However, the minimal data set underlying the findings is archived at the Institute of Sports and Sports Science of the Karlsruhe Institute of Technology (KIT) and can be accessed by interested researchers on site. On-site access should be submitted to the Institute of Sports and Sports Science, Karlsruhe Institute of Technology, Engler-Bunte-Ring 15, 76,131 Karlsruhe, Germany (info@sport.kit.edu).
